# The Development of Genetic Modification Techniques in Intracellular Parasites and Potential Applications to Microsporidia

**DOI:** 10.1371/journal.ppat.1005283

**Published:** 2015-12-31

**Authors:** Aaron W. Reinke, Emily R. Troemel

**Affiliations:** Division of Biological Sciences, Section of Cell and Developmental Biology, University of California-San Diego, San Diego, California, United States of America; Duke University Medical Center, UNITED STATES

## Introduction

Microsporidia constitute a large phylum of eukaryotic obligate intracellular pathogens that can infect a variety of animal hosts. Understanding the biology of microsporidia is severely limited by our current inability to genetically manipulate these parasites. With a growing number of microsporidian genome sequences available and the revolution of successful CRISPR/Cas9 genome editing in virtually every organism tested, the development of DNA transformation techniques would likely lead to rapid and significant progress in understanding microsporidia biology. Here, we outline the challenges in transformation development and review the exciting recent development of DNA transformation and CRISPR/Cas9 genetic manipulation of *Cryptosporidium*, as well as summarize the successful genetic manipulation strategies in other intracellular eukaryotic parasites, including *Plasmodium* and *Toxoplasma*. By providing a list of possible strategies for transforming microsporidia, we hope to facilitate progress in this important area of microbiology and pathogenesis.

## The Importance and Challenge of Developing Genetics in Microsporidia

In order to understand gene function, it is critical to be able to manipulate the gene product. Such manipulation includes both the removal of the whole gene as well as the introduction of specific mutations, which can then be analyzed in vivo for phenotypic consequences. Additionally, it is important to be able to create fusions with epitopes and fluorescent proteins that will allow for the study of protein function in vivo. Unfortunately, such experiments have not been possible for any species of Microsporidia, which constitute a phylum of over 1,400 species of obligate intracellular parasites [1]. This shortcoming has hampered progress in understanding the life cycle, development, and strategies of pathogenesis for these ubiquitous parasites.

There are compelling reasons for dedicating a significant effort toward developing transformation techniques in microsporidia. They can infect a wide range of animal species, many of which are medically and agriculturally important. They are also of great evolutionary interest, being among the earliest branch of sequenced fungi that have intriguing derived characteristics such as an infection apparatus, called a polar tube, and an obligate intracellular lifestyle [1]. Species of microsporidia also have the smallest known eukaryotic genomes, with as few as 1,833 genes in the human-infecting species *Encephalitozoon intestinalis* [2]. This compactness provides both a model of a minimal eukaryotic genome as well as an experimental advantage in that is relatively inexpensive to sequence them and analyze genetic modifications.

A number of independent efforts have been dedicated toward sequencing microsporidia, including the Microsporidian Genomes Consortium at the Broad Institute (http://www.broadinstitute.org/annotation/genome/microsporidia_comparative/MultiHome.html), and >20 genome sequences for several species are now housed at the MicrosporidiaDB website, which is part of EuPathDB eukaryotic pathogens database [3,4]. In addition to genome data, a number of transcriptome studies have defined gene expression in microsporidia at different stages of the microsporidia life cycle [5–7]. From these studies there are candidate regulatory sequences upstream and downstream of highly expressed genes that can be used for driving heterologous expression. With these tools available, and together with the stunning revolution in genome editing due to CRISPR/Cas9-mediated manipulation [8], it is a propitious time to focus significant effort toward developing genetics in microsporidia.

There are a number of considerations in designing a successful approach to genetic modification of an intracellular pathogen. Here, we briefly describe a few key variables to consider in developing DNA transformation for any obligate intracellular organism. First, one must design a transgene construct that will be expressed in the organism of interest. For example, targeting constructs are often designed with regions of upstream and downstream sequence from highly expressed genes of the organism of interest. These regions flank a selection marker that can include drug resistance genes, fluorescent proteins, luminescent proteins, or other reporter enzymes. This expression construct can also be flanked with homologous regions that can aid in integration into a defined site in the genome. After making a targeting construct, the DNA must be introduced into the parasite. There are a number of potential methods for delivery of the DNA, and it is also important to consider whether to introduce the DNA into the parasite in an extracellular stage or an intracellular stage inside of the host. Keeping these issues in mind, we describe below the methods that have been successful for other obligate eukaryotic intracellular parasites and consider possible strategies for successful modification of microsporidia.

## Transformation and Genetics of *Toxoplasma*

The first reported successes in genetically modifying an obligate intracellular eukaryotic pathogen were in the early 1990s with the development of tools for the apicomplexan *Toxoplasma gondii*. The initial breakthrough in *Toxoplasma* genetics was the ability to transiently overexpress reporter constructs. To do this, infective parasites were electroporated with reporter DNA containing the chloramphenicol acetyltransferase (CAT) gene that was flanked by regulatory regions for a highly expressed gene. These transformed parasites were then used to infect human cells, and successful transformation was assessed by measuring CAT enzyme activity [9]. Subsequently, both CAT and an engineered dihydrofolate reductase (DHFR) gene were used as drug-selectable markers, and the stable incorporation of DNA in the form of random integration and homologous recombination was achieved [10,11]. Additional methods for selection were later developed, including other drug-selectable markers, selection based on auxotrophy, and negative selection [10,12–14]. The ability to localize proteins by green fluorescent protein (GFP) fusions was subsequently developed [15]. Another advance was the use of restriction enzyme-mediated integration (REMI), which increases the transformation efficiency and enables stable transfection through nonhomologous integration into the genome [16]. Disruption of the nonhomologous end joining (NHEJ) pathway was shown to greatly increase the efficiency of homologous recombination events, which allows for endogenous modification of gene products [17]. Recently, CRISPR/Cas9 methods have been developed for *Toxoplasma* that allow for enhanced efficiency for targeted modifications [18,19]. These groundbreaking studies in *Toxoplasma* paved the way for developing genetics and transformation in two other apicomplexans, *Plasmodium* and *Cryptosporidium*, as described below.

## Transformation and Genetics of *Plasmodium*

The ability to genetically modify species of *Plasmodium* follows a trajectory similar to the seminal work with *Toxoplasma gondii*. *Plasmodium* species are apicomplexan parasites that are the causative agents of malarial disease. Transient expression of enzymatic activity in *Plasmodium faliciparum* was first achieved by electroporating red blood cells infected with the parasite [20]. Because *Plasmodium* genomes do not encode the genes for canonical NHEJ for DNA repair, integration into the genome has been achieved with homologous recombination (although *Plasmodium* does appear to be capable of an alternative NHEJ pathway for DNA repair [21]). Homologous recombination was used to generate stable transgenes containing engineered DHFR genes that confer resistance to the drug pyrimethamine [22]. Another species of *Plasmodium*, *Plasmodium berghei*, was transformed by electroporation of an infective stage of parasite, and selection of transformed pathogens was performed in mice using a resistant DHFR protein and pyrimethamine [23]. It was further demonstrated that incorporation of GFP could be used in *P*. *berghei* to select for transformed parasites [24]. Recently, CRISPR methods were also demonstrated to work effectively in *Plasmodium* [25,26].

## Transformation and Genetics of *Cryptosporidium*

The most recent example of developing successful genetic manipulation of an obligate intracellular parasite is in another apicomplexan called *Cryptosporidium parvum* [27]. This report represents a systematic tour de force in which the authors developed both transient expression and site-specific integration of this medically significant genus of parasites. First, the researchers developed transient transfection of *Cryptosporidium parvum* in cell culture. They used electroporation to deliver an expression vector containing the nanoluciferase gene, which is a very small and highly luminescent protein that appears to have been key to successfully determining that DNA had been delivered—it was not possible to detect signal with other luminescent or fluorescent proteins. *Cryptosporidium parvum* is propagated in cattle, and researchers delivered this nanoluciferase construct into sporozoites isolated from oocysts, which can be isolated from cattle feces. With this assay in hand, the transgene expression levels were then optimized using different regulatory regions, codon usage, and electroporation conditions. This system was also used to introduce the drug resistance gene *Neo*, which confers resistance to the drug paromomycin that blocks *Cryptosporidium* growth. Next, these researchers developed a method to permanently introduce this transgenic DNA and also modify endogenous loci. Here, they used CRISPR/Cas9 genome editing techniques to enable permanent modification of the genome and stable propagation of transgenic parasites. They took advantage of the fact that *Cryptosporidium* is capable of homologous recombination (and apparently not canonical NHEJ, based on the absence of canonical enzymes [27]), and created transgenes with homology regions upstream and downstream of a nanoluciferase-*Neo* reporter. This transgene was introduced together with DNA encoding Cas9 and a guide RNA to direct the transgene to replace the *Cryptosporidium* thymidine kinase gene. Parasites containing the thymidine kinase locus replaced with the reporter gene were then introduced into mice in order to test the function of thymidine kinase and demonstrate permanent modification of the *Cryptosporidium* genome.

## Strategies and Issues for Developing Transformation in Microsporidia

**Fig 1:**
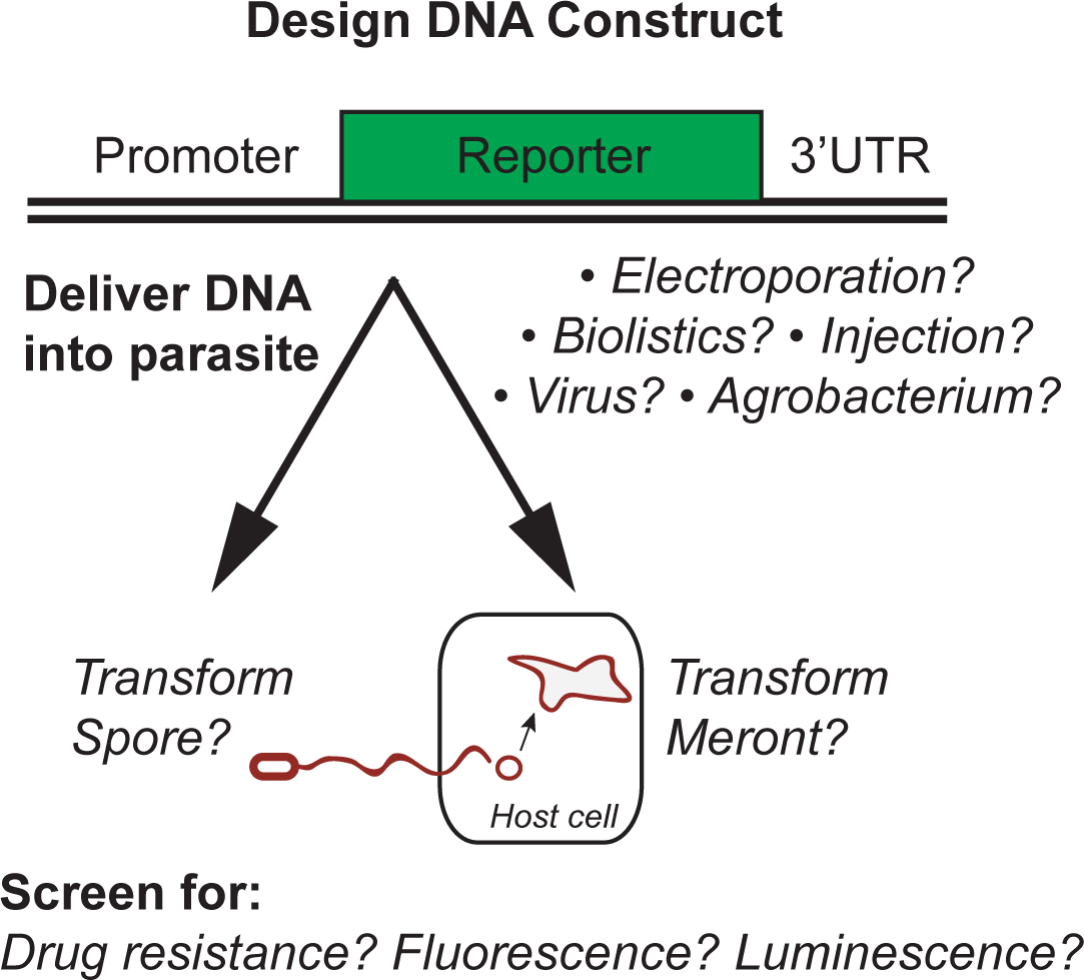
Issues to consider in developing DNA transformation in microsporidia.

The successful examples of developing genomic modifications in other obligate intracellular eukaryotic parasites provide insights and guidance for the possibilities of developing genomic modifications in microsporidia. Here, we consider the key variables in developing DNA transformation for microsporidia: screening method, DNA delivery method, DNA maintenance, and the life stage to be transformed (Fig 1). Similar to the parasites described above, microsporidia are obligate intracellular pathogens, which means they must be grown inside of host cells in order to assess effective incorporation of DNA. Indeed, there are few species outside of the *Encephalitozoon* genus that can be grown in tissue culture—most species actually need to be propagated inside of whole animals. Depending on the species and host, monitoring pathogen growth can be a challenging task. In *Toxoplasma* transformations, pathogen growth can be monitored by identifying plaques, i.e., the absence of growth in a lawn of host cells. Such simple visual assays that rely on host cell health don’t exist for microsporidia. As an alternative, pathogen growth can be successfully monitored in many species by the isolation of spores. One of the most powerful screening methods is to select for drug resistance, which has the benefit of being able to select for very rare events out of a population of untransformed microbes. One drug that is effective against several species of microsporidia is a methionine aminopeptidase inhibitor called fumagillin [28]. The yeast *Saccharomyces cerevisiae* is resistant to fumagillin, which is due to the presence a fumagillin-resistant version of methionine aminopeptidase called MetAP1, which is functionally redundant with the fumagillin-sensitive methionine aminopeptidase MetAP2 [29, 30]. Thus, MetAP1 could potentially be used as a drug resistance marker for microsporidia transformation (Louis Weiss, personal communication, see Acknowledgments). In the absence of visually monitoring pathogen growth, an alternative screening method is to use a fluorescent protein or luminescent protein. An advantage to this screening method is that it does not require efficacy of the drug and function of the drug resistance gene in a novel context to provide resistance against that drug. However, a limitation of fluorescent or luminescent proteins is the amount of time and effort needed to screen and that they are potentially not as sensitive as drug resistance genes in selecting for rare transformation events. Often, as we have described above, combining both drug resistance genes and fluorescent or luminescent proteins together is the most powerful approach.

A second issue to consider in developing transformation in microsporidia is the DNA delivery method. The delivery method appears to be fairly specific to the organism of choice, but options include electroporation, bombardment—also known as biolistics or "gene gun"—chemical transfection, direct injection, *Agrobacterium* (bacterial-mediated) transformation, or viral infection. A third issue to consider is whether the DNA will be delivered in a method that facilitates integration into the genome or if it will leave the DNA in an episomal or extrachromosomal state. Here, one can look at the genome sequence to determine whether there is the presence of enzymes that are important for homologous recombination or NHEJ that will integrate DNA into the genome. This type of analysis has been performed for some species of microsporidia [31,32]. These analyses indicate that microsporidia likely possess the core MRX/MRN enzyme complex that is used for both canonical NHEJ and homologous recombination, but they lack other key enzymes for canonical NHEJ and also lack other key enzymes for homologous recombination [32]. One confounding factor in these studies may be the extreme sequence divergence of microsporidia genomes that can impair the identification of orthologs. If microsporidia truly lack key enzymes for NHEJ, perhaps they use an alternative NHEJ pathway, like *Plasmodium*. Perhaps there is also an alternative pathway for homologous recombination. Overall though, it is unclear from genome gazing whether NHEJ or homologous recombination is a better approach for transforming microsporidia, although presumably microsporidia must have some method for repair of double-stranded DNA breaks.

A fourth issue that is particularly important for developing transformation and genetics in obligate intracellular parasites is which life stage to transform. Microsporidia have a complex life cycle consisting of multiple intracellular stages and an infective extracellular spore form. The intracellular stages have very complex development, and there are limited options for extracting them from cells. Microsporidia have especially intimate interaction with the host cell during their intracellular replication and development. In many species, the replicative stage, called a meront, develops in direct contact with the cytoplasm. In some ways, this structure is similar to a cellular organelle, and thus the strategies that have been successful for transforming mitochondrial and chloroplast genomes (e.g., biolistics for DNA delivery) may be useful for transforming microsporidian meronts inside of host cells. Of note, transformation of the apicomplexans mentioned above was primarily achieved by targeting the extracellular stage that survives outside the host, although initial studies with *Plasmodium falciparum* did successfully electroporate the intracellular stage inside a vacuole [20]. Interestingly, intracellular *Plasmodium* can also be transformed inside of red blood cells by taking up plasmid DNA from the cytoplasm [33]. Thus, in the case of microsporidia, it may be possible to introduce DNA into the host cell cytoplasm, which could then be taken up into the intracellular meront stage. Although the lack of an additional host membrane around the meront of most microsporidian species should be an advantage in terms of DNA delivery, this intimate contact with the host cell is a disadvantage in terms of the difficulty of culturing these meronts outside the host cell. Indeed, there are no reports of culturing meronts that have completed a life cycle and gone on to produce more parasites. The only extracellular stage that survives outside the host is a highly resistant spore that has a thick cell wall. This cell wall may be quite difficult to penetrate using standard electroporation methods. Examples of successful transformation in yeast that have thick cell walls may provide guidance in how to transform microsporidian spores (see further comments below). Also important to note is the fact that the microsporidian spore contains a specialized infection apparatus called a polar tube. This polar tube “fires” in response to the appropriate cue to deliver a parasite cell into the host cell. Thus, the spore is likely quite sensitive to the kinds of treatments used for delivering DNA, such as electroporation and biolistics. These treatments potentially could cause the spore to fire the tube, rendering it noninfectious, although this concern has not been formally demonstrated. This issue could be especially problematic if the selection will occur in an animal instead of a cell line because of the additional demands for viability of a spore in an in vivo environment. Thus, the major options for which life stage to transform are likely to be the extracellular spore form or the meront form within the host cell, each of which provides a unique set of challenges.

A fifth issue to mention is ploidy. The studies described above for apicomplexans involve transforming the haploid stage of parasites, whereas the ploidy of microsporidia has not been well described. However, it is likely they are at least diploid for much of their life cycle in some species [5,34,35]. Transformation of the haploid stage of an organism instead of the diploid state can facilitate genetic analysis because the effects of mutations can be assessed without the masking effects of a wild-type gene on the homologous chromosome present in a diploid. However, the haploid state can be a disadvantage for genetic analysis if the mutation has deleterious effects that make it difficult to isolate a viable mutant. Notably, genetic analysis by transgenesis in diploid organisms may not be as challenging as it once was, given the recent demonstration that multiple homozygous knockouts can be obtained with high efficiency using genetically encoded Cas9 in one generation in studies with mice [36] and flies [37]. In terms of generating gain-of-function changes, such as the introduction of drug-resistant genes or fluorescent proteins, the diploid nature of microsporidia should not pose much of an issue, particularly if there are selection methods or if genetically encoded Cas9 is used.

## Concluding Remarks

The impressive work on transformation methods in the apicomplexans described above is likely to provide a powerful blueprint and proof-of-principle that obligate intracellular pathogens can be transformed and transgenic parasites can be selected in whole animals, though there are other sources of guidance to be considered. While microsporidia are obligate intracellular pathogens, they are quite distinct from apicomplexans, and as the earliest branching group of fungi they share characteristics with yeast such as chitinous spores [5]. Because of this similarity, approaches that have been successfully adopted for a number of yeast and filamentous fungal species might deserve prioritization [38]. Of note, pathogenic yeast such as *Cryptococcus neoformans* are transformed much less efficiently than the genetic model yeast *Saccharomyces cerevisiae*, but with optimization there have been improvements [39]. In particular, *Cryptococcus* and other fungi with tough cell walls have been successfully transformed with biolistics as well as by *Agrobacterium*-mediated transformation. Another lesson from *Cryptococcus* transformation has been that large homology regions upstream and downstream of targeting constructs is key to successful homologous recombination. In addition to these lessons from fungi, there is guidance and inspiration in the rapid advances of techniques for genetic manipulation of intracellular bacterial pathogens [40,41].

While it is difficult to predict which transformation method will be successful for microsporidia, here we make a few recommendations. First off, while transformation could be developed by measuring an intermediate step of the process, such as delivery of the DNA, we believe that effort is better directed toward optimizing an endpoint. Our reasoning is that optimization of an intermediate step (for example, assessing DNA delivery with the use of fluorescent DNA oligos), will not necessarily optimize the endpoint, which is to achieve efficient genomic modification and gene expression. Second, we propose that because of its relative simplicity and the recent success in *Cryptosporidium*, effort should be focused on transforming a luminescent protein such as nanoluciferase or one of the newer, brighter fluorescent protein variants such as mNeonGreen [42] or mRuby2 [43]. While introducing a drug resistance gene can provide sensitivity for low frequency events, it demands that the drug be nearly 100% effective to avoid false positive transformants and that the drug resistance gene function in an untested physiological context. Third, in terms of how the DNA will be integrated into the microsporidian genome, we favor using regions of homology in the targeting construct to promote homologous recombination, given its success in transformation of fungi and apicomplexans. Fourth, in terms of life stage to transform, we favor targeting the intracellular meront stage, perhaps using biolistics, although the delivery method will depend on the host cell to be targeted. Here, our reasoning is that the extracellular spore stage has an extremely tough wall, together with a polar tube that might be quite sensitive to mechanical disruption that would occur through DNA delivery, and thus the meront may more likely be transformable while maintaining viability. Overall, however, development of genetic modification methods in microsporidia is likely to be species-specific, and successful approaches will vary even between different species of microsporidia. Thus, we believe the approach that is likely to be the most successful is an empirical one of testing multiple reporters, promoter sequences, and different microsporidian species. We have highlighted above examples of successful approaches in other eukaryotic pathogens and general principles that can be followed in order to spur development of genetics in microsporidia and help us learn about this important but poorly understood group of obligate intracellular parasites.
